# A Small-Angle Neutron Scattering Methodology for Quantitative Characterization of Channel Width in Gamma Matrix Phase

**DOI:** 10.3390/nano15201581

**Published:** 2025-10-16

**Authors:** Zhong Chen, Tianfu Li, Erdong Wu, Xiaoming Du, Shaohua Zhang, Shibo Yan, Zijun Wang, Kai Sun, Dongfeng Chen

**Affiliations:** 1 China Institute of Atomic Energy, Beijing 102413, China; chenz@ciae.ac.cn (Z.C.); 15901460287@163.com (S.Y.); wzj2345@163.com (Z.W.); ksun@sina.com (K.S.); 2 Key Lab for Neutron Scattering Technology and Application, China National Nuclear Corporation (CNNC), Beijing 102413, China; 3 Institute of Metal Research, Chinese Academy of Sciences, Shenyang 110016, China; ewu@imr.ac.cn (E.W.); zhangshaohua@imr.ac.cn (S.Z.); 4 College of Materials Science and Engineering, Shenyang Ligong University, Shenyang 110159, China; du511@163.com

**Keywords:** small-angle neutron scattering, γ matrix channel, superalloy, lamellar model

## Abstract

This study establishes a robust small-angle neutron scattering (SANS) methodology for the quantitative characterization of γ matrix channel widths in the nickel-based single-crystal superalloy DD10. By combining SANS with TEM analyses and modeling the one-dimensional SANS data via a polydisperse lamellar model, we accurately determined the channel width distribution across macroscopic sample volumes. In the virgin state, the mean channel widths were nearly isotropic, measuring 17.8 ± 0.1 nm along [002] and 20.5 ± 0.1 nm along [020]. After standard heat treatment (solution and two-step aging), significant anisotropic coarsening was observed, with widths increasing to 36.8 ± 0.2 nm along [002] and 28.0 ± 0.1 nm along [020], indicating stress-free rafting. Elemental mapping revealed substantial redistribution of key alloying elements: Al content in γ′ precipitates increased by 2.6 at.%, while Cr in the γ channels rose by 5.9 at.%. These quantitative results demonstrate that SANS provides reliable, bulk-statistical insights into nanoscale channel geometry, highlighting its critical role in influencing elemental diffusion kinetics and microstructural evolution during thermal exposure.

## 1. Introduction

Nickel-based superalloys are the backbone materials for high-temperature components in aerospace and energy-generation systems, such as jet engine turbine blades and disks, due to their exceptional strength, creep resistance, and oxidation stability at extreme conditions [1]. This performance critically relies on their characteristic two-phase microstructure: coherent, cuboidal precipitates of the ordered γ’ phase (Ni_3_Al-type, L1_2_ structure) embedded within a solid solution γ matrix [2]. The continuous network of γ matrix separating the γ’ precipitates, known as the γ matrix channels, plays a pivotal role. These channels act as the primary pathways for dislocation motion during deformation [3] and crucially, as the dominant routes for elemental diffusion between γ and γ’ phases during high-temperature service [4,5]. The width of these γ channels is therefore a fundamental microstructural parameter governing both mechanical properties (e.g., creep resistance) and microstructural evolution (e.g., γ’ coarsening, rafting) [6,7].

Because the γ channels constitute the dominant diffusion pathways between γ and γ’, their width directly sets the effective diffusion length and, consequently, the local solute equilibrium at the γ/γ’ interface. Recent experimental work by Saksena et al. [8] demonstrates that even when temperature and bulk composition are kept constant, variations of only a few tens of nanometers in channel width noticeably alter the compositional trajectory and coarsening rate of γ’ precipitates. Although current phase-field and Kampmann–Wagner Numerical (KWN) simulations still treat interface energy, diffusion coefficients and lattice misfit as the primary input parameters [9], an incorrectly characterized initial channel geometry propagates an indirect but measurable error into the predicted temporal evolution of precipitate size and volume fraction. Therefore, when constructing the initial digital microstructure or validating simulation results against experiment, the channel width distribution should be quantified with an uncertainty budget; doing so improves the reliability of both alloy-design predictions and remaining-life assessments [8,9].

Accurately quantifying the γ channel width, particularly its distribution and evolution under thermal exposure, is essential for predicting alloy performance and lifetime. However, current characterization techniques face significant limitations. Transmission electron microscopy (TEM), including high-resolution (HRTEM) techniques, provides high spatial resolution but suffers from limited field-of-view and inherent projection artifacts, making statistically robust measurements of channel widths across representative sample volumes extremely laborious and prone to sampling bias [10,11]. While atom probe tomography (APT) offers near-atomic resolution and direct chemical information at the γ/γ’ interface, its analyzed volume is minuscule (typically <0.01 µm^3^), severely restricting its ability to characterize the broader channel network structure and width distribution [12]. These limitations highlight a critical gap: the lack of a technique capable of providing quantitative, statistically significant data on γ channel widths across macroscopic sample volumes.

Furthermore, understanding how γ channel width influences elemental diffusion behavior within the channels is vital for modeling microstructural evolution. While diffusion coefficients in bulk γ are reasonably well-established [8], diffusion within the confined geometry of narrow γ channels, potentially influenced by proximity to interfaces and local chemical gradients, remains less understood. A key controversy exists regarding the relative importance of channel width versus interfacial chemical gradients in controlling element transport rates between γ and γ’ phases during coarsening. Some models suggest diffusion kinetics are primarily governed by the matrix composition gradient [13,14], while others emphasize the critical role of physical channel width in constraining diffusion paths and potentially altering effective diffusivities [15]. Resolving this requires experimental data directly correlating channel geometry with diffusion behavior.

The interplay between mechanical properties and diffusion processes further underscores the need for advanced characterization. For example, applied stress during service not only influences dislocation activity within the channels but can also induce directional diffusion, leading to the formation of γ’ rafts [16,17]. The initial channel width is a pre-existing microstructural condition that mediates the alloy’s response to such thermomechanical loading [18]. Without a clear understanding of the baseline channel structure, decoupling the effects of stress, temperature, and microstructure on performance becomes exceedingly difficult.

To address these challenges, we introduced Small-Angle Neutron Scattering (SANS) as a quantitative method for characterizing γ matrix channel widths. SANS probes density or composition fluctuations on length scales typically ranging from ~1 nm to several hundred nanometers [19], ideally matching the dimensions of γ channels in superalloys. Its key advantages include: (1) bulk sampling (analyzing mm^3^-scale volumes), providing unparalleled statistical representation; (2) non-destructive nature, enabling in situ studies; and (3) sensitivity to nanoscale structural periodicity inherent in the γ/γ’ microstructure [20]. While SANS has been successfully applied to study γ’ precipitate size, volume fraction, and morphology [21,22,23,24], its potential for directly quantifying γ channel widths has been largely unexplored and underutilized.

We anticipate that the SANS methodology developed here will serve as a complementary tool to existing techniques, bridging the gap between the high-resolution, small-volume data from TEM/APT and the large-volume, statistical representation needed for confident materials characterization [25]. This approach is particularly suited for tracking temporal changes in the microstructure during in situ heating experiments, offering a dynamic view of evolution processes that traditional post-mortem analysis cannot capture [26].

The primary aims of this work are twofold:

To develop and validate a robust SANS-based methodology for the quantitative determination of γ matrix channel widths and their distributions in Ni-based superalloys.

To utilize this approach to investigate the correlation between γ channel width and the diffusion behavior of key alloying elements (e.g., Al, Cr, W) within these channels during thermal aging.

## 2. Materials and Methods 

The first-generation Ni-based superalloy DD10 samples were provided by China’s Institute of Metal [27]. One sample underwent standard heat treatment, while the other remained virgin. Both were prepared as 1 mm-thick, 15 mm-diameter circular slices for SANS measurements, with TEM samples made after neutron experiments.

The samples are the first-generation Ni-based single crystal superalloy DD10 which contains 13Cr, 4Co, 13.2 (Al+Ti+Ta), 6 (W+Mo), with minor C and B, and Ni in balance (weight percent), and is supplied by the Institute of Metal, China Academic of Science. One sample received the standard heat treatment, comprising a two-step solution treatment, first at 1493 K for 3 h and followed by 1523 K for 3 h both in air and then a two-step aging treatment, first at 1373 K for 4 h and subsequently at 1173 K for 24 h, both followed by air-cooling [28]. The other one remains in virgin state. The two samples were made into a circular slice with a thickness of 1 mm and a diameter of about 15 mm for SANS measurements. The normal direction of the circular slice was along the [200] direction. TEM samples were made from the same samples after the neutron experiment.

**Figure 1:**
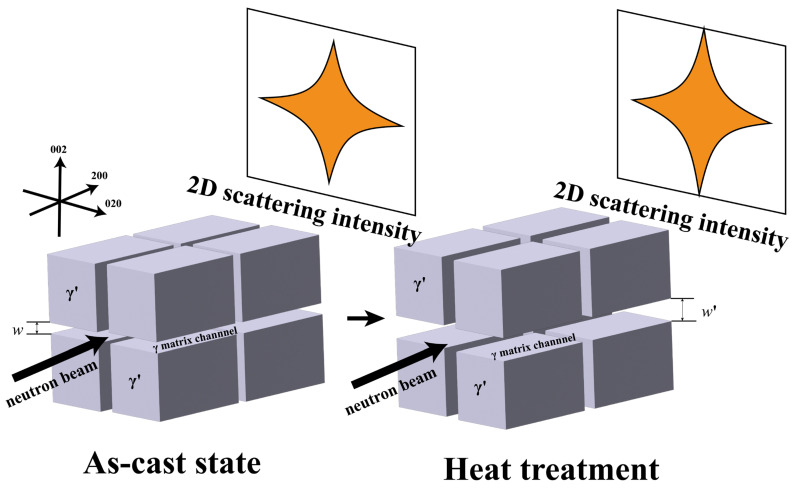
Schematic illustration of the SANS methodology for quantifying γ matrix channel width. The indices (002, 200, 020) denote crystallographic directions within the cubic lattice of the superalloy, as defined by Miller indices.

Figure 1 shows a schematic of the SC superalloy structure, cubic γ’ precipitates in γ matrix channels, and corresponding SANS data patterns. The neutron beam is along the [200] direction, with a cross-shaped scattering pattern on the 2D detector. Sector integration for 1D data extraction was performed with an angular window of ±15° centered on the [002] and [020] directions. The 1D SANS data can be analyzed using a lamellar model [29].

For the lamellar structure with poly-dispersed width, the scattering intensity *I*(*Q*) can be expressed as:

(1) $$I(Q)=\mathrm{scale}\int_{w}\frac{2\pi P(Q,w)}{Q^{2}w}f(w)dw+\mathrm{background}$$

where scale stands for a scale factor, the background is for scattering background, *P*(*Q,w*) is the lamellar form factor, and *f*(*w*) is the width distribution function.

*P*(*Q,w*) can be written as:

(2) $$P(Q,w)=\frac{2\Delta \rho^{2}}{Q^{2}}(1-\cos(Qw))=\frac{4\Delta \rho^{2}}{Q^{2}}\sin^{2}(\frac{Qw}{2})$$

where Δρ stands for neutron scattering length density contrast between γ and γ’, and *w* stands for γ matrix channel width.

(3) $$f(w)=\frac{1}{\mathrm{Norm}}\frac{1}{w\sigma }\exp\left(-\frac{1}{2}{\left(\frac{\ln(w)-\ln(w_{\mathrm{med}})}{\sigma }\right)}^{2}\right)$$

A lognormal distribution function for the channel width is found to fit the experimental data well. [14] Norm is the normalization factor, $w_{\mathrm{med}}$ is the median value of the lognormal distribution, and σ is a parameter describing the width of the underlying normal distribution.

TEM and HRTEM (The original manufacturer of the FEI Tecnai G20 transmission electron microscope (TEM) and its high-resolution variant (HRTEM) is FEI Company, and the equipment was sourced from Eindhoven, The Netherlands.) were used to observe the metallographic microstructures of the samples. Thin foils were prepared by cutting, mechanically grinding to ~60 μm, and thinning with a twin-jet polishing system at 20 °C using a 5% perchloric acid and 95% ethanol solution.

## 3. Results

### 3.1. Microstructural Characterization via TEM and SANS

**Figure 2:**
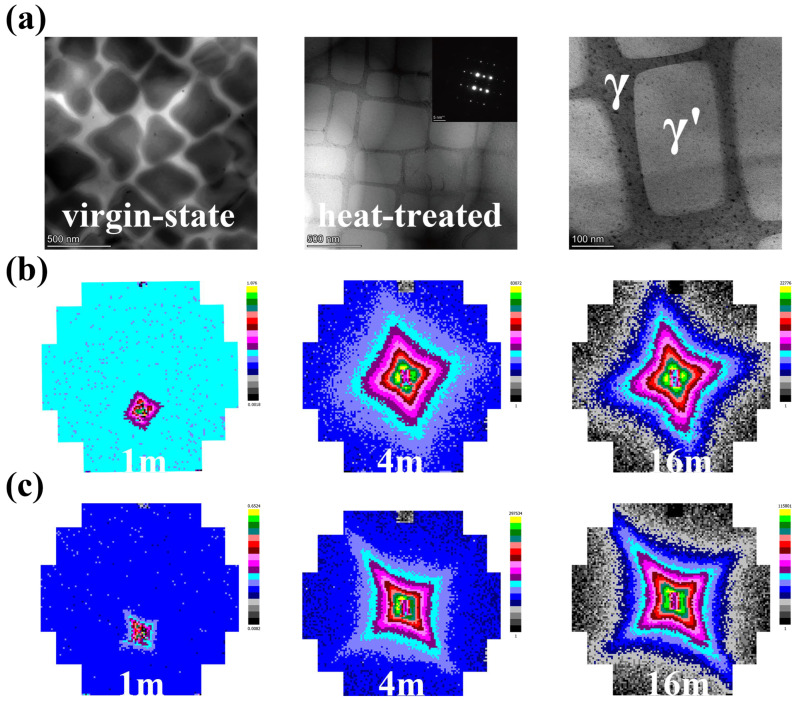
TEM and the 2D SANS data, (**a**) TEM of virgin state and heat-treated sample; (**b**) SANS pattern of the virgin state sample; (**c**) SANS pattern of 1173K heat-treated sample.

Transmission electron microscopy (TEM) analysis revealed distinct microstructural differences between the virgin and heat-treated DD10 superalloy samples (Figure 2a). The virgin microstructure exhibited irregularly shaped γ’ precipitates, forming a complex γ matrix channel network primarily oriented perpendicularly. In contrast, the heat-treated sample (1173K aged) displayed predominantly regular, cuboidal γ’ precipitates within the γ matrix, alongside smaller secondary γ’ precipitates present within the γ channels.

Small-Angle Neutron Scattering (SANS) experiments were conducted at HZB, wherein two-dimensional Small-Angle Neutron Scattering (2D SANS) patterns corroborated these microstructural observations (Figure 2b,c). The virgin sample produced an anisotropic cross-shaped pattern, with comparable scattering intensity along the [020] and [002] directions, consistent with the observed irregular precipitate morphology. The heat-treated sample exhibited a more pronounced anisotropic cross-pattern, with distinct differences in scattering intensity between the [020] and [002] directions. This anisotropy indicates a directional coarsening or “rafting” of the γ/γ’ microstructure occurring during the heat treatment process. Analysis confirmed that the high-Q region of the SANS data primarily arose from the scattering contribution of the small secondary γ’ precipitates and background, not the γ matrix channels themselves.

### 3.2. Quantitative Analysis of γ Matrix Channel Width

**Figure 3:**
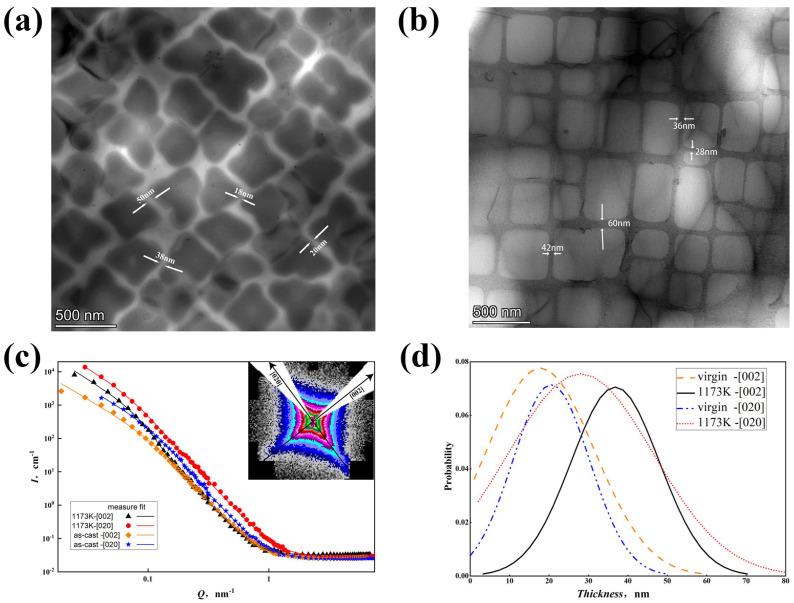
TEM results and SANS fitting results: (**a**) Virgin state sample TEM results with several marked channel widths at different places, (**b**) heat-treatment sample TEM results with several marked channel widths at different places, (**c**) model fitting results of the 1D SANS data by sector integral of the direction [002] and [020], and (**d**) the channel width distribution according to the model fitting results.

Quantitative analysis of γ matrix channel widths was performed using both TEM measurements and SANS data modeling (Figure 3). TEM analysis of the virgin sample (Figure 3a) yielded channel widths ranging from approximately 20 nm to 60 nm. TEM analysis of the heat-treated sample (Figure 3b) showed a similar range but suggested potential differences among different crystallographic directions.

SANS data analysis provided a statistically robust quantification of channel width distributions. The 1D SANS intensity profiles, obtained by sector integration along the [002] and [020] directions, were successfully fitted using a polydisperse lamellar model (Figure 3c, Equations (1)–(3)). A lognormal distribution function provided an excellent fit to the experimental data, yielding the most probable γ channel width (W) and distribution parameters (σ) for each direction and sample condition (Figure 3d, Table 1).

Virgin Sample: SANS analysis yielded consistent channel widths along both [020] and [002] directions: W [020] = 20.5 nm and W [002] = 17.8 nm. The irregular precipitate morphology results in a relatively narrow mean γ channel width.

Heat-Treated Sample (1173K): SANS revealed a clear anisotropy in channel width: W [020] = 28.0 nm and W [002] = 36.8 nm. This signifies directional coarsening (rafting) where the channels widened preferentially along the [020] direction compared to [002], leading to a larger average channel width and a narrower distribution along [020]. The most probable widths from SANS (Table 1) were qualitatively consistent with the ranges observed by TEM.

Figure 3 shows quantitative analysis of γ matrix channel width from TEM and SANS results. TEM results display channel widths around 20–60 nm, while 1D SANS data fits the polydisperse lamellar model well (Figure 3c). Lognormal distribution fitting (Figure 3d) and most probable thickness width (Table 1) show SANS and TEM results qualitatively agree. SANS measures bulk samples, so discussions focus on SANS results.

For virgin samples, the γ matrix channel width shows little difference in [020] and [200] directions, around 20 nm. The precipitation phase’s irregular shape resembles a quasicrystal structure, resulting in a narrower mean γ matrix channel. Heat-treated samples show larger average γ matrix channel width in [020] than [200] and narrower distribution, indicating rafting during heat treatment, which accelerates under high temperatures and stress.

The γ matrix channel widening during creep has been investigated by several other groups in recent decades [15], and the dependence of matrix channel width on temperature, stress, and time has been carefully analyzed and different models were proposed. Stress has been recognized to play an important role. However, the effect of heat treatment without external stress on the γ matrix channel width has not been a focus of discussions in these reports. Most of these studies were based on TEM and simulations. Our SANS results here clearly show that even though there is no stress applied to the sample during the heat process, the γ matrix channel widening behaves differently along the two directions. The reason could be the different residual stress levels along different directions in the measured sample. In addition, the lattice misfit of γ channel and γ’ precipitates may be different along two directions, which could result in anisotropic diffusion.

### 3.3. Elemental Partitioning Behavior and Evolution upon Heat Treatment

**Figure 4:**
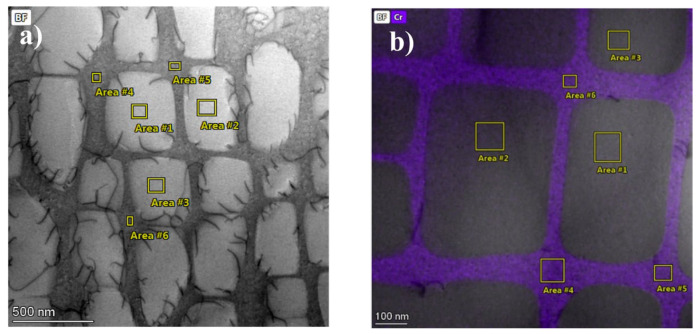
Test areas. (**a**) Virgin state sample test area, (**b**) heat-treatment sample test area.

**Figure 5:**
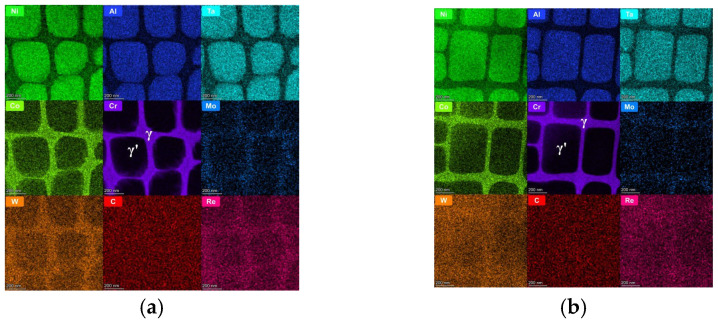
Elemental mapping. (**a**) Virgin state sample, (**b**) heat-treated sample.

Elemental distribution analysis across designated microstructural regions (γ’ precipitates: Regions 1, 2, 3; γ matrix channels: Regions 4, 5, 6) Figure 4a,b revealed distinct partitioning preferences for Ni, Al, Ta, Co, Cr, Mo, W, C, and Re in both sample states, as illustrated in Figure 5a,b. Ni, Al, and Ta demonstrated significant enrichment within the γ’ precipitates, while Co, Cr, Mo, W, and Re were primarily partitioned to the γ matrix channels. Carbon exhibited a dispersed presence throughout both phases. Ni and Al consistently displayed higher concentrations within the γ’ precipitates compared to the γ matrix channels, with Cr exhibiting the inverse partitioning behavior, favoring the matrix channels. Within the γ’ precipitates, Ta and W concentrations were comparable; however, Ta concentration in the γ matrix was consistently lower than within γ’.

**Figure 6:**
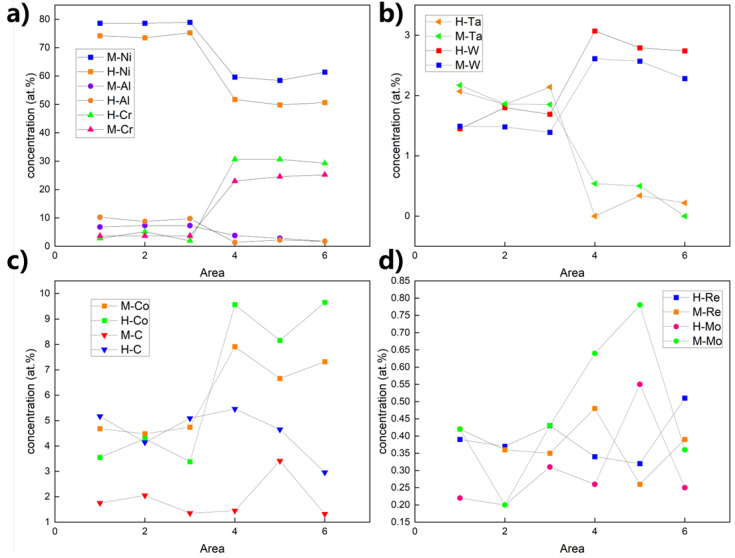
Elemental distribution. (**a**) Ni, Al, Cr; (**b**) Ta, W; (**c**) Co, C; and (**d**) Mo, Re.

Figure 6 details the compositional evolution following thermal exposure. Heat treatment induced a relative decrease in Ni and Al concentrations specifically within the γ matrix channels. The spatial distribution profile of Ta was notably altered by the heat treatment. Carbon concentration increased measurably within both phases after aging. Cobalt partitioning shifted significantly, characterized by decreased concentration within the γ’ precipitates and a concomitant increase within the γ matrix channels. The distribution of Re became more homogeneous throughout the microstructure after heat treatment. Furthermore, trace elements Re and Mo exhibited a consistent trend towards reduced concentrations within both phases following thermal exposure. These synergistic alterations in elemental distributions are anticipated to significantly influence diffusion kinetics and microstructural stability.

Quantitative analysis of the regions marked in Figure 5 and Table 2 (areas 1–3: γ’ precipitates; areas 4–6: γ matrix channels) provides a statistical measure of this evolution. In the virgin state (M), the partitioning behavior is evident: the γ’ precipitates were enriched in Ni (~78.7 at.%), Al (~7.1 at.%), and Ta (~2.0 at.%), while the γ channels were enriched in Cr (~24.2 at.%) and Co (~7.3 at.%). Heat treatment (H) significantly amplified this partitioning. The concentration of the γ’-former Al increased by over 35% within the precipitates (to ~9.7 at.%), while the γ-former Cr increased by nearly 25% within the channels (to ~30.2 at.%). Concurrently, Ni content decreased markedly in both phases, most notably within the γ channels (from ~59.8 at.% to ~50.6 at.%, a reduction of ~9.2 at.%). This quantitative data confirms that the heat treatment drove the system towards a more stable thermodynamic equilibrium, resulting in a sharper chemical contrast between the γ and γ’ phases. The widening of the γ channels, as quantified by SANS, facilitates this large-scale redistribution of elements, directly linking the nanoscale geometrical evolution to the macroscopic chemical kinetics.

## 4. Discussion

### 4.1. SANS as a Quantitative Tool for Channel Width Characterization

This study successfully establishes SANS as a quantitative methodology for characterizing γ matrix channel widths in Ni-based superalloys. The key advantage of SANS lies in its bulk sampling capability (analyzing mm^3^ volumes), providing statistically significant data on channel width distributions that are difficult to achieve with highly localized techniques like TEM or APT. The excellent fit of the polydisperse lamellar model to the anisotropic SANS data (Figure 3c,d) is consistent with its applicability for extracting quantitative geometric parameters (median width, distribution breadth) of the γ channel network. The qualitative agreement between SANS-derived most probable widths and TEM measurements suggests that the SANS approach yields reliable results, while highlighting SANS’s superior capability for capturing representative averages across the macroscopic sample volume.

### 4.2. Anisotropic Channel Widening and Rafting Mechanisms

A significant finding from the SANS analysis is the clear anisotropy in γ matrix channel width observed in the heat-treated sample (W[020]= 28.0 nm vs. W[002]= 36.8 nm). This directional difference is a signature of microstructural rafting. While rafting is commonly associated with the application of external stress during high-temperature creep, our observations suggest that anisotropic channel evolution can occur even during standard heat treatment involving aging without an externally applied stress.

This finding is consistent with, but does not uniquely prove, the influence of intrinsic factors driving the rafting anisotropy:

Residual Stress: Directional variations in residual stress within the sample, potentially arising from thermal gradients during processing or cooling, may provide the driving force for directional diffusion and anisotropic coarsening.

Anisotropic Lattice Misfit: Differences in the lattice misfit parameter along the [020] and [002] directions might exist. Anisotropic misfit strain fields could generate directional elastic strain fields that favor diffusion and coarsen along specific crystallographic axes, thereby widening channels preferentially in certain directions. The observed widening along [020] and relative narrowing along [002] is therefore consistent with rafting behavior driven by such internal or local strain fields although direct measurement of such fields was not performed.

### 4.3. Implications for Diffusion and Microstructural Evolution

The quantification of γ channel width and its anisotropy has direct implications for understanding elemental diffusion kinetics within the constrained geometry of the channels, a key factor controlling microstructural evolution (e.g., γ’ coarsening, rafting) and high-temperature properties. Diffusion coefficients measured in bulk γ may not accurately represent diffusion within nanoscale channels. Our SANS results provide direct experimental evidence of significant channel width variations (e.g., 28.0 nm vs. 36.8 nm in the heat-treated sample) and distributions.

Narrower channels, particularly those below a critical width, may impose geometric constraints on diffusion paths (“confinement effect”). This confinement is expected to reduce the effective diffusivity of alloying elements (Al, Cr, W) compared to their bulk values, as hypothesized in theoretical models [30,31]. The anisotropy in channel width further implies that diffusion kinetics will be directionally dependent. Our findings are therefore consistent with models where the physical geometry (width and its distribution) of the γ channels plays a dominant role in governing diffusion-limited processes like coarsening and rafting, alongside the influence of compositional gradients [13]. The SANS methodology developed here offers a powerful tool to directly correlate channel geometry parameters with diffusion measurements (e.g., via tracer studies or interdiffusion experiments) to resolve the ongoing controversy regarding the relative importance of channel width versus interfacial chemistry gradients.

### 4.4. Comparison with Prior Work

While numerous studies have documented γ channel widening during creep (under applied stress) and linked it to stress direction [32], our SANS results uniquely demonstrate that anisotropic widening may occur during stress-free heat treatment. This observation suggests that intrinsic factors may contribute to rafting-like channel evolution. The ability of SANS to detect and quantify this subtle, yet crucial, microstructural anisotropy underlines its sensitivity and value for fundamental microstructural characterization beyond techniques primarily sensitive to precipitate characteristics.

## 5. Conclusions

This study establishes a robust SANS methodology for quantifying γ matrix channel widths in Ni-based superalloys. The key conclusions are:

A quantitative approach combining SANS with a polydisperse lamellar model was successfully developed, providing bulk, statistical characterization of γ channel widths that complements localized techniques like TEM.

In the virgin state, the γ channels were nearly isotropic, with mean widths of 17.8 ± 0.1 nm along [002] and 20.5 ± 0.1 nm along [020].

Standard heat treatment induced significant anisotropic coarsening. Channel widths increased to 36.8 ± 0.2 nm along [002] and 28.0 ± 0.1 nm along [020], indicating stress-free rafting.

Elemental mapping revealed substantial redistribution following annealing: Al content in γ′ precipitates increased by 2.60 at%, while Cr in the γ channels rose by 5.94 at%.

The observed anisotropy is attributed to intrinsic factors such as residual stress and anisotropic lattice misfit, highlighting the importance of channel geometry in influencing diffusion kinetics and microstructural evolution.

This SANS methodology provides a reliable, statistical tool for future studies on microstructural evolution under thermal and mechanical loading.

## Figures and Tables

**Table 1:** The polydisperse lamellar model fit results.

Name	Most Probable Thickness (nm)
[002] virgin	17.8 ± 0.1
[020] virgin	20.5 ± 0.1
[002] Heat-treated	36.8 ± 0.2
[020] Heat-treated	28.0 ± 0.1

**Table 2:** Average elemental composition (at.%) in γ’ and γ.

Element	Virgin(M)		Heat-Treated (H)		Change (H − M)	
	γ’	γ	γ’	γ	γ’	γ
Ni	78.69	59.82	73.29	50.59	−5.40	−9.23
Al	7.14	2.01	9.74	1.66	+2.60	−0.35
Cr	3.68	24.24	2.96	30.18	−0.72	+5.94
Co	4.63	7.30	3.81	9.14	−0.82	+1.84
Ta	1.96	0.35	2.02	0.11	+0.06	−0.24
W	1.45	2.49	1.65	2.87	+0.20	+0.38

## Data Availability

The data that support the findings of this study are available from the corresponding author upon reasonable request.

## Abbreviations

The following abbreviations are used in this manuscript:

APT	Atom Probe Tomography
SANS	Small-Angle Neutron Scattering
TEM	Transmission Electron Microscopy
γ	gamma matrix phase
γ’	gamma prime precipitate phase
HRTEM	High-Resolution Transmission Electron Microscopy
DD10	A specific first-generation Ni-based single-crystal superalloy
L1_2_	A type of ordered crystal structure (e.g., Ni_3_Al)
SC	Single Crystal
Q	Scattering vector
I(Q)	Scattering intensity
Δρ	Neutron scattering length density contrast
w	Width of the γ matrix channel
w_med	Median channel width (from lognormal fit)
σ	Width parameter of the lognormal distribution
[hkl]	Miller indices denoting a crystallographic direction
(hkl)	Miller indices denoting a crystallographic plane
nm	Nanometer
